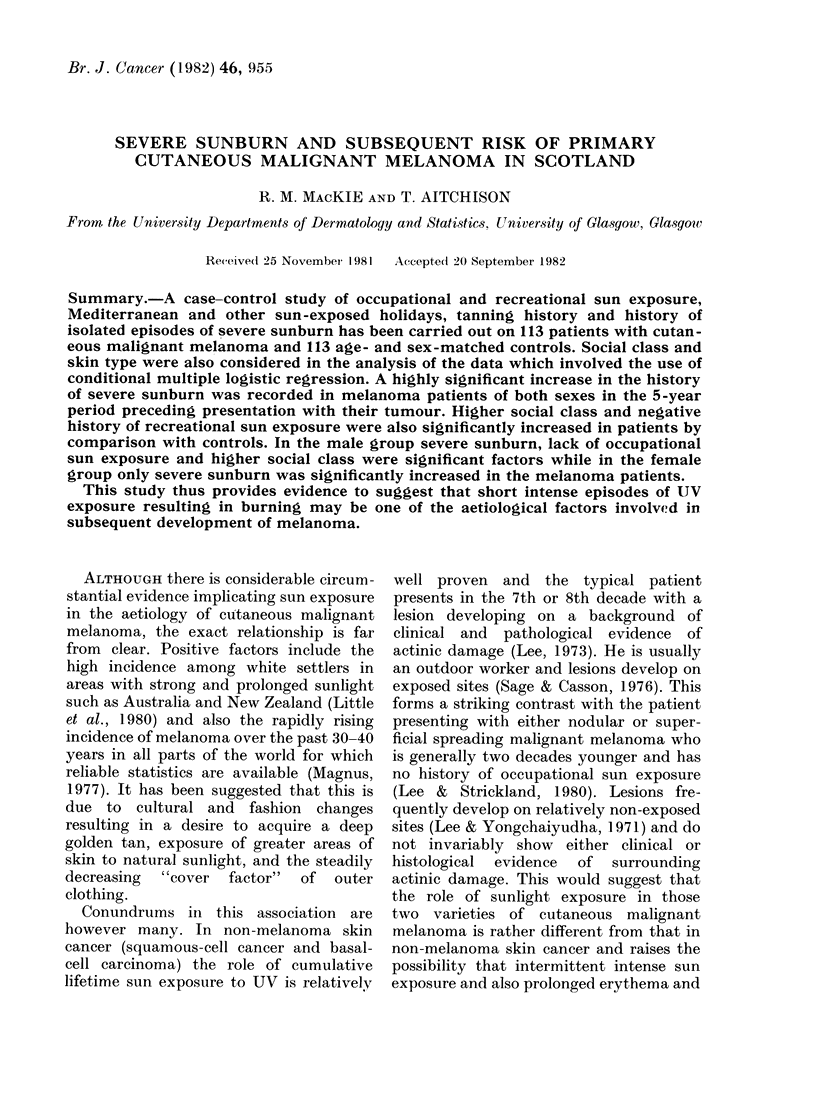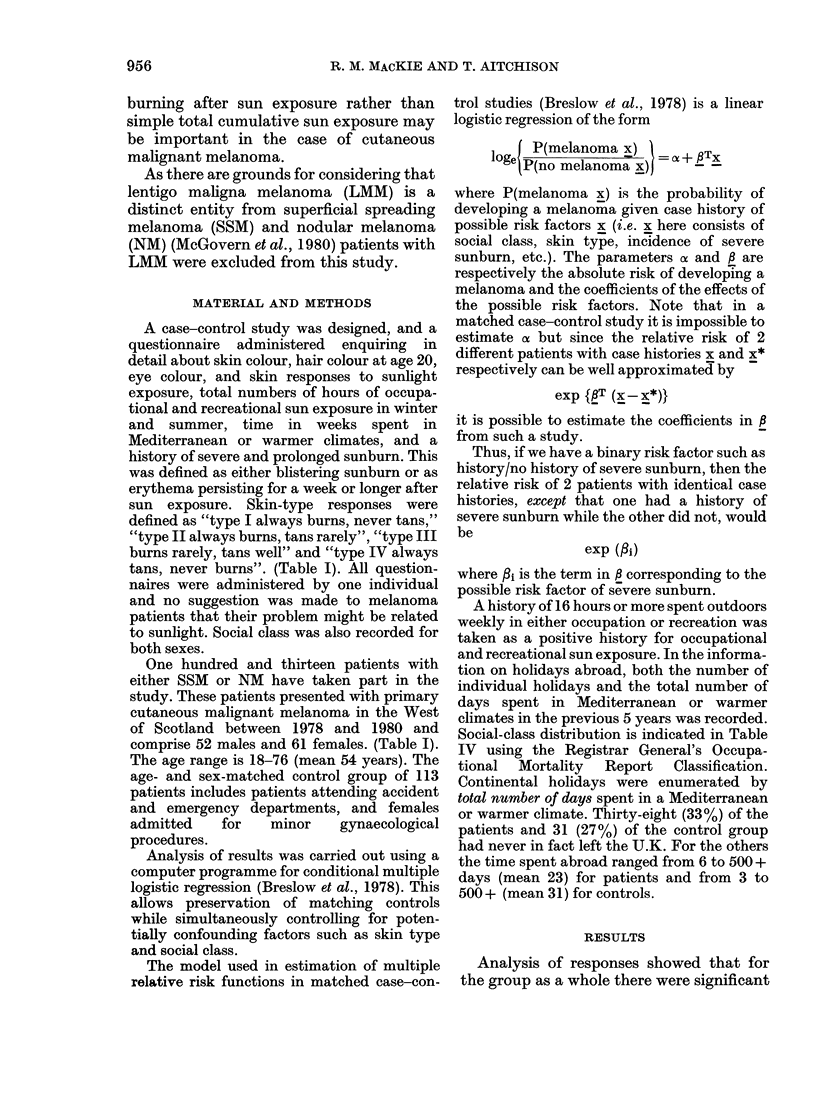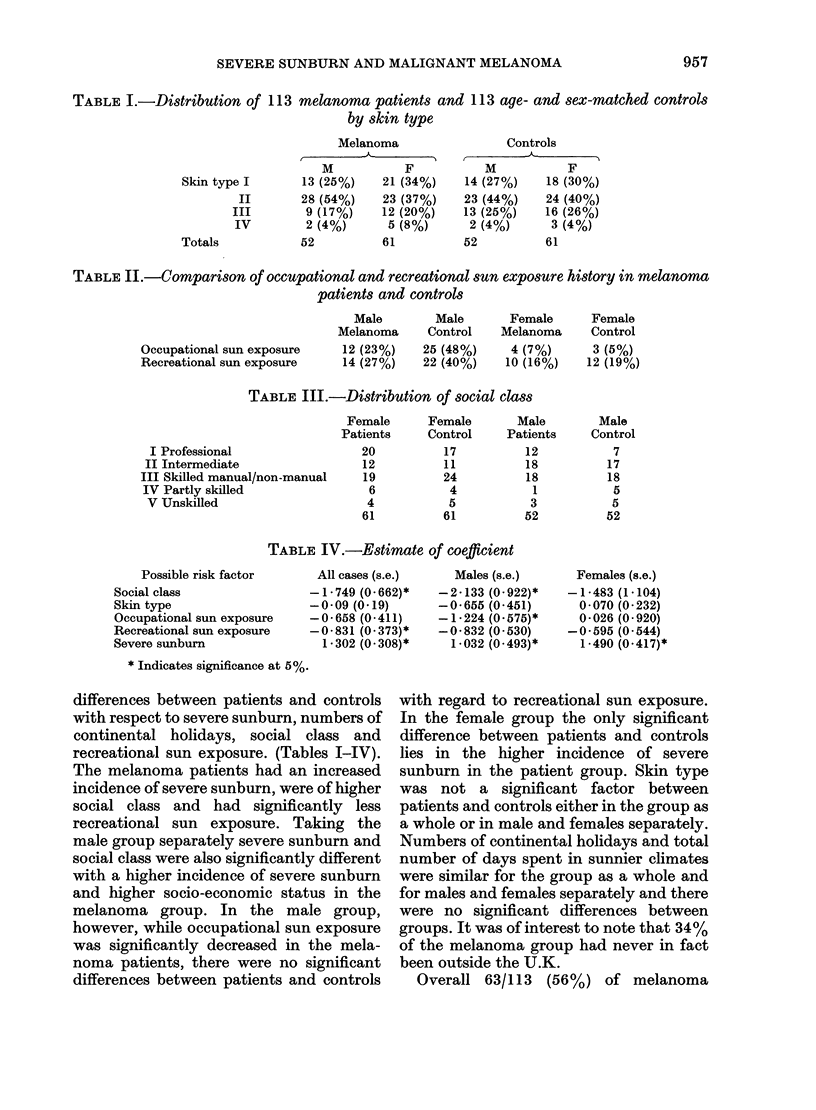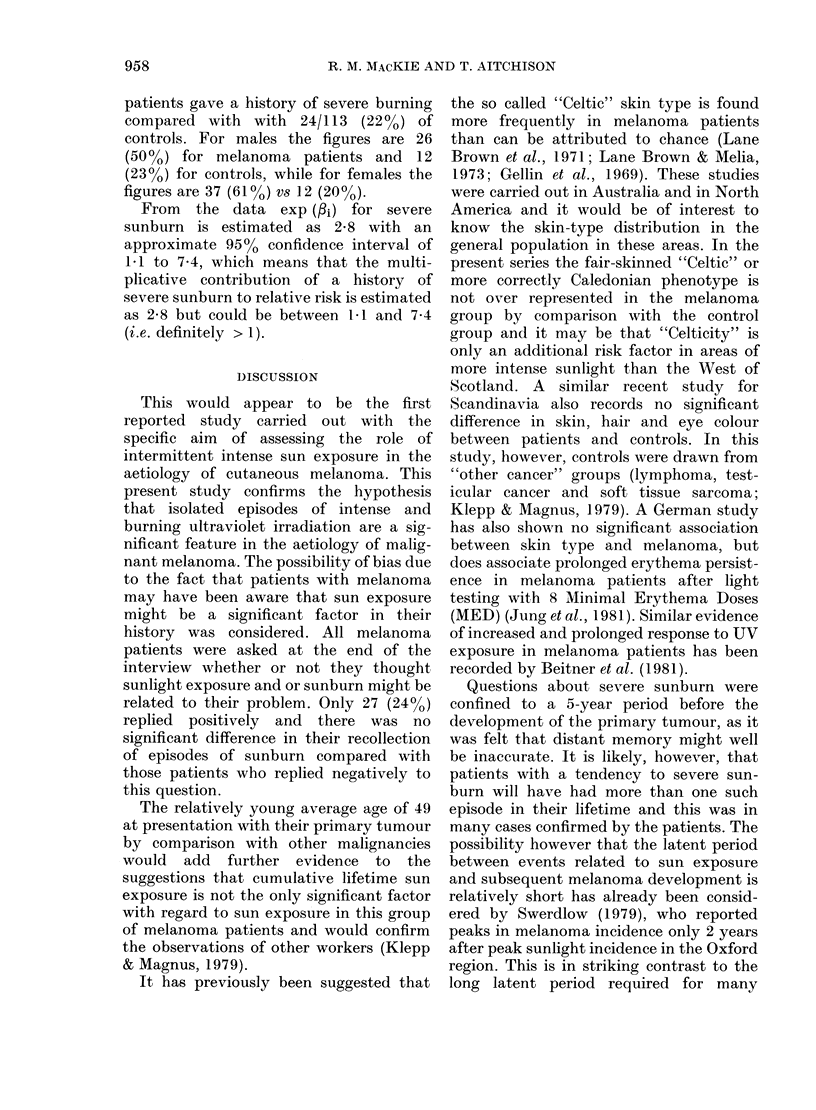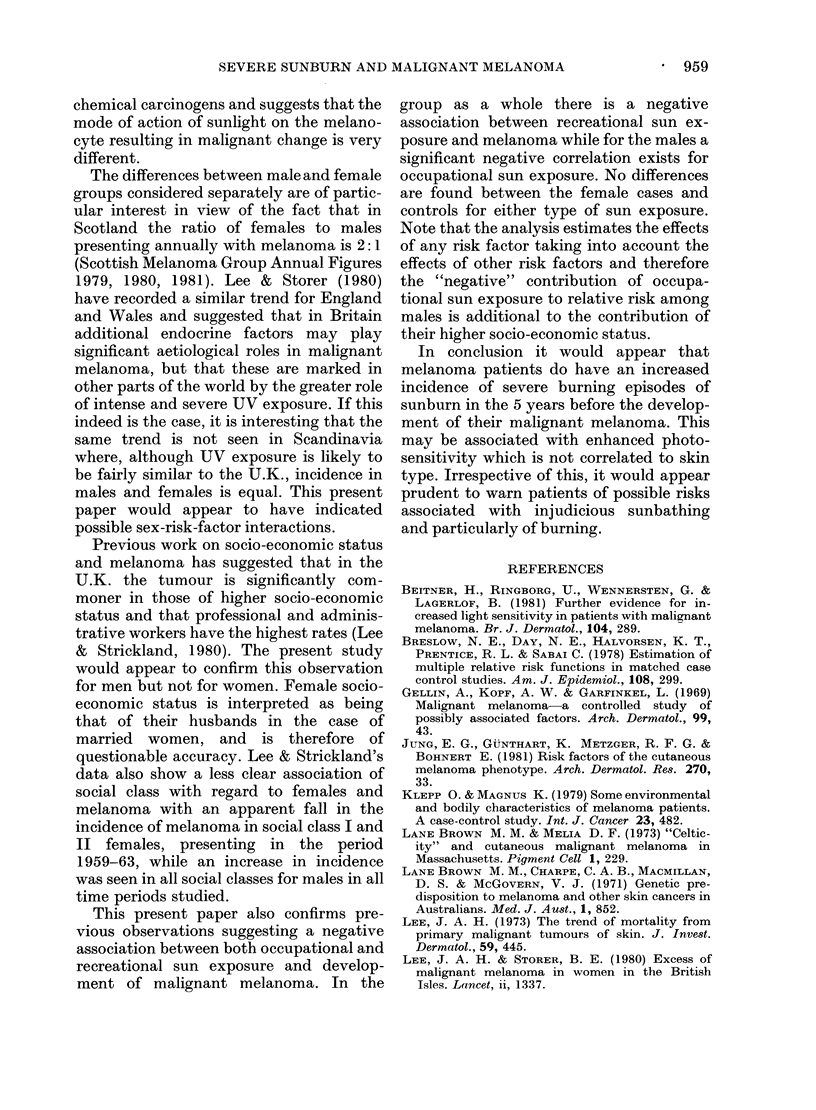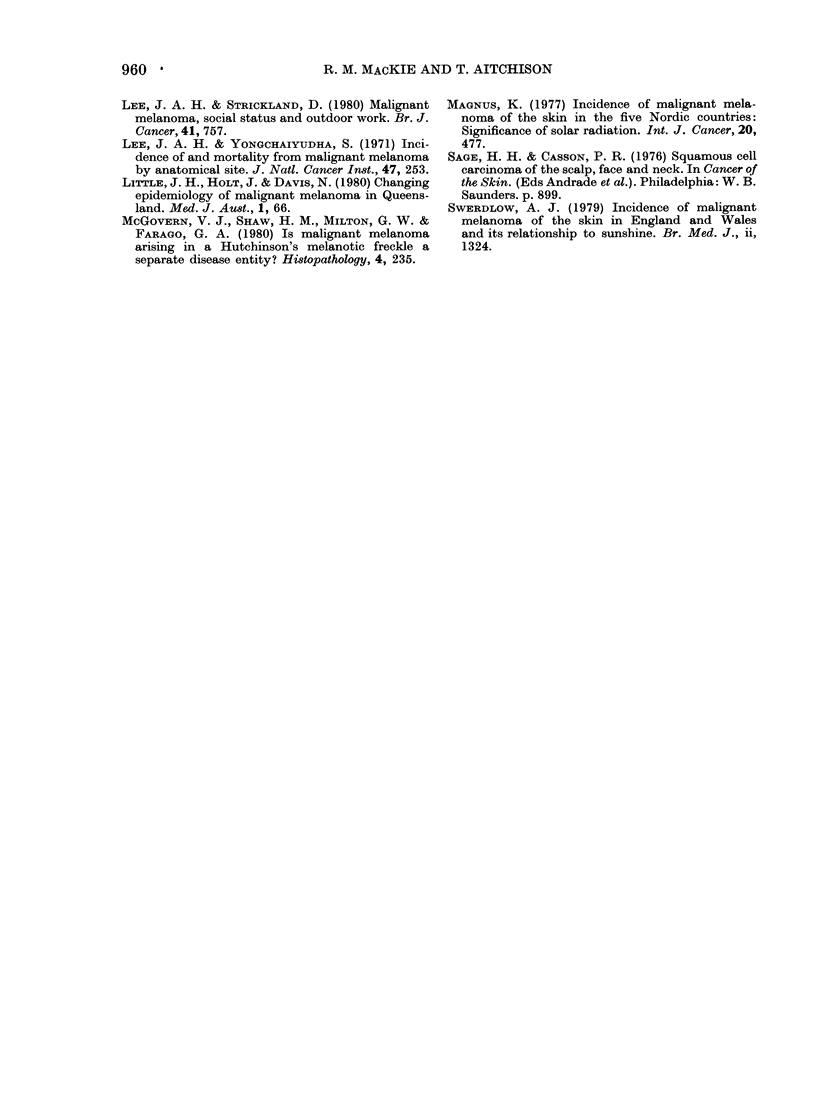# Severe sunburn and subsequent risk of primary cutaneous malignant melanoma in scotland.

**DOI:** 10.1038/bjc.1982.307

**Published:** 1982-12

**Authors:** R. M. MacKie, T. Aitchison

## Abstract

A case-control study of occupational and recreational sun exposure, Mediterranean and other sun-exposed holidays, tanning history and history of isolated episodes of severe sunburn has been carried out on 113 patients with cutaneous malignant melanoma and 113 age- and sex-matched controls. Social class and skin type were also considered in the analysis of the data which involved the use of conditional multiple logistic regression. A highly significant increase in the history of severe sunburn was recorded in melanoma patients of both sexes in the 5-year period preceding presentation with their tumour. Higher social class and negative history of recreational sun exposure were also significantly increased in patients by comparison with controls. In the male group severe sunburn, lack of occupational sun exposure and higher social class were significant factors while in the female group only severe sunburn was significantly increased in the melanoma patients. This study thus provides evidence to suggest that short intense episodes of UV exposure resulting in burning may be one of the aetiological factors involved in subsequent development of melanoma.


*Br. J. Cancer* (1982) **46,** 955

### SEVERE SUNBURN AND SUBSEQUENT RISK OF PRIMARY CUTANEOUS MALIGNANT MELANOMA IN SCOTLAND

R. M. MACKIE AND T. AITCHISON

*From the University Departments of Dermatology and Statistics, University of Glasgow, Glasgow*

Received 25 November 1981 Accepted 20 September 1982

**Summary.—A case-control study of occupational and recreational sun exposure, Mediterranean and other sun-exposed holidays, tanning history and history of isolated episodes of severe sunburn has been carried out on 113 patients with cutaneous malignant melanoma and 113 age- and sex-matched controls. Social class and skin type were also considered in the analysis of the data which involved the use of conditional multiple logistic regression. A highly significant increase in the history of severe sunburn was recorded in melanoma patients of both sexes in the 5-year period preceding presentation with their tumour. Higher social class and negative history of recreational sun exposure were also significantly increased in patients by comparison with controls. In the male group severe sunburn, lack of occupational sun exposure and higher social class were significant factors while in the female group only severe sunburn was significantly increased in the melanoma patients.**

**This study thus provides evidence to suggest that short intense episodes of UV exposure resulting in burning may be one of the aetiological factors involved in subsequent development of melanoma.**

ALTHOUGH there is considerable circumstantial evidence implicating sun exposure in the aetiology of cutaneous malignant melanoma, the exact relationship is far from clear. Positive factors include the high incidence among white settlers in areas with strong and prolonged sunlight such as Australia and New Zealand (Little *et al.*, 1980) and also the rapidly rising incidence of melanoma over the past 30–40 years in all parts of the world for which reliable statistics are available (Magnus, 1977). It has been suggested that this is due to cultural and fashion changes resulting in a desire to acquire a deep golden tan, exposure of greater areas of skin to natural sunlight, and the steadily decreasing "cover factor" of outer clothing.

Conundrums in this association are however many. In non-melanoma skin cancer (squamous-cell cancer and basal-cell carcinoma) the role of cumulative lifetime sun exposure to UV is relatively well proven and the typical patient presents in the 7th or 8th decade with a lesion developing on a background of clinical and pathological evidence of actinic damage (Lee, 1973). He is usually an outdoor worker and lesions develop on exposed sites (Sage & Casson, 1976). This forms a striking contrast with the patient presenting with either nodular or superficial spreading malignant melanoma who is generally two decades younger and has no history of occupational sun exposure (Lee & Strickland, 1980). Lesions frequently develop on relatively non-exposed sites (Lee & Yongchaiyudha, 1971) and do not invariably show either clinical or histological evidence of surrounding actinic damage. This would suggest that the role of sunlight exposure in those two varieties of cutaneous malignant melanoma is rather different from that in non-melanoma skin cancer and raises the possibility that intermittent intense sun exposure and also prolonged erythema and burning after sun exposure rather than simple total cumulative sun exposure may be important in the case of cutaneous malignant melanoma.

As there are grounds for considering that lentigo maligna melanoma (LMM) is a distinct entity from superficial spreading melanoma (SSM) and nodular melanoma (NM) (McGovern *et al.*, 1980) patients with LMM were excluded from this study.

### MATERIAL AND METHODS

A case–control study was designed, and a questionnaire administered enquiring in detail about skin colour, hair colour at age 20, eye colour, and skin responses to sunlight exposure, total numbers of hours of occupational and recreational sun exposure in winter and summer, time in weeks spent in Mediterranean or warmer climates, and a history of severe and prolonged sunburn. This was defined as either blistering sunburn or as erythema persisting for a week or longer after sun exposure. Skin-type responses were defined as "type I always burns, never tans," "type II always burns, tans rarely", "type III burns rarely, tans well" and "type IV always tans, never burns". (Table I). All questionnaires were administered by one individual and no suggestion was made to melanoma patients that their problem might be related to sunlight. Social class was also recorded for both sexes.

One hundred and thirteen patients with either SSM or NM have taken part in the study. These patients presented with primary cutaneous malignant melanoma in the West of Scotland between 1978 and 1980 and comprise 52 males and 61 females. (Table I). The age range is 18–76 (mean 54 years). The age- and sex-matched control group of 113 patients includes patients attending accident and emergency departments, and females admitted for minor gynaecological procedures.

Analysis of results was carried out using a computer programme for conditional multiple logistic regression (Breslow *et al.*, 1978). This allows preservation of matching controls while simultaneously controlling for potentially confounding factors such as skin type and social class.

The model used in estimation of multiple relative risk functions in matched case–control studies (Breslow *et al.*, 1978) is a linear logistic regression of the form

$$\log_e\left\{\frac{P(\text{melanoma } \underline{x})}{P(\text{no melanoma } \underline{x})}\right\} = \alpha + \underline{\beta}^T \underline{x}$$

where P(melanoma $\underline{x}$) is the probability of developing a melanoma given case history of possible risk factors $\underline{x}$ (*i.e.* $\underline{x}$ here consists of social class, skin type, incidence of severe sunburn, etc.). The parameters $\alpha$ and $\underline{\beta}$ are respectively the absolute risk of developing a melanoma and the coefficients of the effects of the possible risk factors. Note that in a matched case–control study it is impossible to estimate $\alpha$ but since the relative risk of 2 different patients with case histories $\underline{x}$ and $\underline{x}^*$ respectively can be well approximated by

$$\exp\{\underline{\beta}^T(\underline{x} - \underline{x}^*)\}$$

it is possible to estimate the coefficients in $\underline{\beta}$ from such a study.

Thus, if we have a binary risk factor such as history/no history of severe sunburn, then the relative risk of 2 patients with identical case histories, *except* that one had a history of severe sunburn while the other did not, would be

$$\exp(\beta_i)$$

where $\beta_i$ is the term in $\underline{\beta}$ corresponding to the possible risk factor of severe sunburn.

A history of 16 hours or more spent outdoors weekly in either occupation or recreation was taken as a positive history for occupational and recreational sun exposure. In the information on holidays abroad, both the number of individual holidays and the total number of days spent in Mediterranean or warmer climates in the previous 5 years was recorded. Social-class distribution is indicated in Table IV using the Registrar General's Occupational Mortality Report Classification. Continental holidays were enumerated by *total number of days* spent in a Mediterranean or warmer climate. Thirty-eight (33%) of the patients and 31 (27%) of the control group had never in fact left the U.K. For the others the time spent abroad ranged from 6 to 500+ days (mean 23) for patients and from 3 to 500+ (mean 31) for controls.

### RESULTS

Analysis of responses showed that for the group as a whole there were significant

TABLE I.—*Distribution of* 113 *melanoma patients and* 113 *age- and sex-matched controls by skin type*

| | Melanoma | | Controls | |
|---|---|---|---|---|
| | M | F | M | F |
| Skin type I | 13 (25%) | 21 (34%) | 14 (27%) | 18 (30%) |
| II | 28 (54%) | 23 (37%) | 23 (44%) | 24 (40%) |
| III | 9 (17%) | 12 (20%) | 13 (25%) | 16 (26%) |
| IV | 2 (4%) | 5 (8%) | 2 (4%) | 3 (4%) |
| Totals | 52 | 61 | 52 | 61 |

TABLE II.—*Comparison of occupational and recreational sun exposure history in melanoma patients and controls*

| | Male Melanoma | Male Control | Female Melanoma | Female Control |
|---|---|---|---|---|
| Occupational sun exposure | 12 (23%) | 25 (48%) | 4 (7%) | 3 (5%) |
| Recreational sun exposure | 14 (27%) | 22 (40%) | 10 (16%) | 12 (19%) |

TABLE III.—*Distribution of social class*

| | Female Patients | Female Control | Male Patients | Male Control |
|---|---|---|---|---|
| I Professional | 20 | 17 | 12 | 7 |
| II Intermediate | 12 | 11 | 18 | 17 |
| III Skilled manual/non-manual | 19 | 24 | 18 | 18 |
| IV Partly skilled | 6 | 4 | 1 | 5 |
| V Unskilled | 4 | 5 | 3 | 5 |
| | 61 | 61 | 52 | 52 |

TABLE IV.—*Estimate of coefficient*

| Possible risk factor | All cases (s.e.) | Males (s.e.) | Females (s.e.) |
|---|---|---|---|
| Social class | −1·749 (0·662)* | −2·133 (0·922)* | −1·483 (1·104) |
| Skin type | −0·09 (0·19) | −0·655 (0·451) | 0·070 (0·232) |
| Occupational sun exposure | −0·658 (0·411) | −1·224 (0·575)* | 0·026 (0·920) |
| Recreational sun exposure | −0·831 (0·373)* | −0·832 (0·530) | −0·595 (0·544) |
| Severe sunburn | 1·302 (0·308)* | 1·032 (0·493)* | 1·490 (0·417)* |

* Indicates significance at 5%.

differences between patients and controls with respect to severe sunburn, numbers of continental holidays, social class and recreational sun exposure. (Tables I–IV). The melanoma patients had an increased incidence of severe sunburn, were of higher social class and had significantly less recreational sun exposure. Taking the male group separately severe sunburn and social class were also significantly different with a higher incidence of severe sunburn and higher socio-economic status in the melanoma group. In the male group, however, while occupational sun exposure was significantly decreased in the melanoma patients, there were no significant differences between patients and controls with regard to recreational sun exposure. In the female group the only significant difference between patients and controls lies in the higher incidence of severe sunburn in the patient group. Skin type was not a significant factor between patients and controls either in the group as a whole or in male and females separately. Numbers of continental holidays and total number of days spent in sunnier climates were similar for the group as a whole and for males and females separately and there were no significant differences between groups. It was of interest to note that 34% of the melanoma group had never in fact been outside the U.K.

Overall 63/113 (56%) of melanoma patients gave a history of severe burning compared with with 24/113 (22%) of controls. For males the figures are 26 (50%) for melanoma patients and 12 (23%) for controls, while for females the figures are 37 (61%) *vs* 12 (20%).

From the data exp ($\beta_i$) for severe sunburn is estimated as 2·8 with an approximate 95% confidence interval of 1·1 to 7·4, which means that the multiplicative contribution of a history of severe sunburn to relative risk is estimated as 2·8 but could be between 1·1 and 7·4 (*i.e.* definitely $>1$).

### DISCUSSION

This would appear to be the first reported study carried out with the specific aim of assessing the role of intermittent intense sun exposure in the aetiology of cutaneous melanoma. This present study confirms the hypothesis that isolated episodes of intense and burning ultraviolet irradiation are a significant feature in the aetiology of malignant melanoma. The possibility of bias due to the fact that patients with melanoma may have been aware that sun exposure might be a significant factor in their history was considered. All melanoma patients were asked at the end of the interview whether or not they thought sunlight exposure and or sunburn might be related to their problem. Only 27 (24%) replied positively and there was no significant difference in their recollection of episodes of sunburn compared with those patients who replied negatively to this question.

The relatively young average age of 49 at presentation with their primary tumour by comparison with other malignancies would add further evidence to the suggestions that cumulative lifetime sun exposure is not the only significant factor with regard to sun exposure in this group of melanoma patients and would confirm the observations of other workers (Klepp & Magnus, 1979).

It has previously been suggested that the so called "Celtic" skin type is found more frequently in melanoma patients than can be attributed to chance (Lane Brown *et al.*, 1971; Lane Brown & Melia, 1973; Gellin *et al.*, 1969). These studies were carried out in Australia and in North America and it would be of interest to know the skin-type distribution in the general population in these areas. In the present series the fair-skinned "Celtic" or more correctly Caledonian phenotype is not over represented in the melanoma group by comparison with the control group and it may be that "Celticity" is only an additional risk factor in areas of more intense sunlight than the West of Scotland. A similar recent study for Scandinavia also records no significant difference in skin, hair and eye colour between patients and controls. In this study, however, controls were drawn from "other cancer" groups (lymphoma, testicular cancer and soft tissue sarcoma; Klepp & Magnus, 1979). A German study has also shown no significant association between skin type and melanoma, but does associate prolonged erythema persistence in melanoma patients after light testing with 8 Minimal Erythema Doses (MED) (Jung *et al.*, 1981). Similar evidence of increased and prolonged response to UV exposure in melanoma patients has been recorded by Beitner *et al.* (1981).

Questions about severe sunburn were confined to a 5-year period before the development of the primary tumour, as it was felt that distant memory might well be inaccurate. It is likely, however, that patients with a tendency to severe sunburn will have had more than one such episode in their lifetime and this was in many cases confirmed by the patients. The possibility however that the latent period between events related to sun exposure and subsequent melanoma development is relatively short has already been considered by Swerdlow (1979), who reported peaks in melanoma incidence only 2 years after peak sunlight incidence in the Oxford region. This is in striking contrast to the long latent period required for many chemical carcinogens and suggests that the mode of action of sunlight on the melanocyte resulting in malignant change is very different.

The differences between male and female groups considered separately are of particular interest in view of the fact that in Scotland the ratio of females to males presenting annually with melanoma is 2:1 (Scottish Melanoma Group Annual Figures 1979, 1980, 1981). Lee & Storer (1980) have recorded a similar trend for England and Wales and suggested that in Britain additional endocrine factors may play significant aetiological roles in malignant melanoma, but that these are marked in other parts of the world by the greater role of intense and severe UV exposure. If this indeed is the case, it is interesting that the same trend is not seen in Scandinavia where, although UV exposure is likely to be fairly similar to the U.K., incidence in males and females is equal. This present paper would appear to have indicated possible sex-risk-factor interactions.

Previous work on socio-economic status and melanoma has suggested that in the U.K. the tumour is significantly commoner in those of higher socio-economic status and that professional and administrative workers have the highest rates (Lee & Strickland, 1980). The present study would appear to confirm this observation for men but not for women. Female socio-economic status is interpreted as being that of their husbands in the case of married women, and is therefore of questionable accuracy. Lee & Strickland's data also show a less clear association of social class with regard to females and melanoma with an apparent fall in the incidence of melanoma in social class I and II females, presenting in the period 1959–63, while an increase in incidence was seen in all social classes for males in all time periods studied.

This present paper also confirms previous observations suggesting a negative association between both occupational and recreational sun exposure and development of malignant melanoma. In the group as a whole there is a negative association between recreational sun exposure and melanoma while for the males a significant negative correlation exists for occupational sun exposure. No differences are found between the female cases and controls for either type of sun exposure. Note that the analysis estimates the effects of any risk factor taking into account the effects of other risk factors and therefore the "negative" contribution of occupational sun exposure to relative risk among males is additional to the contribution of their higher socio-economic status.

In conclusion it would appear that melanoma patients do have an increased incidence of severe burning episodes of sunburn in the 5 years before the development of their malignant melanoma. This may be associated with enhanced photosensitivity which is not correlated to skin type. Irrespective of this, it would appear prudent to warn patients of possible risks associated with injudicious sunbathing and particularly of burning.

### REFERENCES

BEITNER, H., RINGBORG, U., WENNERSTEN, G. & LAGERLOF, B. (1981) Further evidence for increased light sensitivity in patients with malignant melanoma. *Br. J. Dermatol.*, **104**, 289.

BRESLOW, N. E., DAY, N. E., HALVORSEN, K. T., PRENTICE, R. L. & SABAI C. (1978) Estimation of multiple relative risk functions in matched case control studies. *Am. J. Epidemiol.*, **108**, 299.

GELLIN, A., KOPF, A. W. & GARFINKEL, L. (1969) Malignant melanoma—a controlled study of possibly associated factors. *Arch. Dermatol.*, **99**, 43.

JUNG, E. G., GÜNTHART, K. METZGER, R. F. G. & BOHNERT E. (1981) Risk factors of the cutaneous melanoma phenotype. *Arch. Dermatol. Res.* **270**, 33.

KLEPP O. & MAGNUS K. (1979) Some environmental and bodily characteristics of melanoma patients. A case-control study. *Int. J. Cancer* **23**, 482.

LANE BROWN M. M. & MELIA D. F. (1973) "Celticity" and cutaneous malignant melanoma in Massachusetts. *Pigment Cell* **1**, 229.

LANE BROWN M. M., CHARPE, C. A. B., MACMILLAN, D. S. & MCGOVERN, V. J. (1971) Genetic predisposition to melanoma and other skin cancers in Australians. *Med. J. Aust.*, **1**, 852.

LEE, J. A. H. (1973) The trend of mortality from primary malignant tumours of skin. *J. Invest. Dermatol.*, **59**, 445.

LEE, J. A. H. & STORER, B. E. (1980) Excess of malignant melanoma in women in the British Isles. *Lancet*, ii, 1337.

LEE, J. A. H. & STRICKLAND, D. (1980) Malignant melanoma, social status and outdoor work. *Br. J. Cancer*, **41**, 757.

LEE, J. A. H. & YONGCHAIYUDHA, S. (1971) Incidence of and mortality from malignant melanoma by anatomical site. *J. Natl. Cancer Inst.*, **47**, 253.

LITTLE, J. H., HOLT, J. & DAVIS, N. (1980) Changing epidemiology of malignant melanoma in Queensland. *Med. J. Aust.*, **1**, 66.

MCGOVERN, V. J., SHAW, H. M., MILTON, G. W. & FARAGO, G. A. (1980) Is malignant melanoma arising in a Hutchinson's melanotic freckle a separate disease entity? *Histopathology*, **4**, 235.

MAGNUS, K. (1977) Incidence of malignant melanoma of the skin in the five Nordic countries: Significance of solar radiation. *Int. J. Cancer*, **20**, 477.

SAGE, H. H. & CASSON, P. R. (1976) Squamous cell carcinoma of the scalp, face and neck. In *Cancer of the Skin.* (Eds Andrade *et al.*). Philadelphia: W. B. Saunders. p. 899.

SWERDLOW, A. J. (1979) Incidence of malignant melanoma of the skin in England and Wales and its relationship to sunshine. *Br. Med. J.*, ii, 1324.